# Skin marker placement by technologist prior to knee MRI helps identify clinically relevant pathologies

**DOI:** 10.1186/s12891-017-1876-7

**Published:** 2017-12-15

**Authors:** Vibhor Wadhwa, Eric Weissman, Daichi Hayashi, Yin Xi, Avneesh Chhabra

**Affiliations:** 1 Department of Radiology, University of Arkansas for Medical Sciences, Little Rock, AR USA; 2 Department of Radiology, UT Southwestern Medical Center, Dallas, TX USA; 3 Department of Radiology, Boston University School of Medicine, Boston, MA USA; 4 Department of Radiology, Stony Brook Medicine, Stony Brook, NY USA; 5 Department of Orthopaedic Surgery, UT Southwestern Medical Center, 5323 Harry Hines Blvd, Dallas, TX 75390-9178 USA; 6 Adjunct faculty, Department of Radiology, Johns Hopkins University, Baltimore, USA

**Keywords:** Knee MRI, MRI skin marker, Meniscus tear, Ligament tear, Cartilage defect

## Abstract

**Background:**

Majority of musculoskeletal cross-sectional imaging requests have a non-revealing and non-specific clinical history of pain. However, the location of pain is very relevant towards arriving at a specific orthopedic diagnosis. The purpose of this research was to study the impact of skin marker placement and training of technologists prior to knee MRI in detection of clinically important findings.

**Methods:**

Total 200 consecutive left knee MRIs were evaluated before and after technologist training with regards to marker placement at the site of clinical symptoms or palpable finding. Marker location in relation to the knee was recorded and important findings were classified as correlated important finding, non-correlated important finding, other compartment important finding in non-correlated cases, and diffuse abnormality, i.e. tri-compartmental cartilage defects in both correlated and non-correlated cases. Differences among scans before and after technologist training were analyzed.

**Results:**

The marker placement was observed in higher proportion of patients in post-training scans (78% vs 60%, *p* = 0.00). The most common location of the marker was in anterior or anterolateral knee (32% and 34% cases, respectively). The marker-important finding correlation was also higher post training, but not statistically significant (53% versus 38%, *p* = 0.57). Important findings correlated with the marker in more than 50% of the scans in the post-training set.

**Conclusion:**

Marker placement can aid in detection of clinically important imaging finding and technologist training aids in increased rates of marker placement and improved correlation.

## Background

In our tertiary care practice, majority of orthopedic cross-sectional study orders have a non-revealing and non-specific (in terms of location) history of pain. However, it is well known that site of pain e.g. joint line tenderness is quiet relevant in arriving at diagnosis of orthopaedic conditions, such as meniscus tear [1, 2]. Similarly, many joint diagnoses based on history and examinations reflect the site of patient’s symptoms. Musculoskeletal radiologists reading these joint MRIs and particularly those using a structured reporting template find many related and unrelated findings with respect to the patient’s presentation. The radiologist may not have access to the patient’s chart during study readout or even with chart access, extracting relevant history and examination takes time and reduces the efficiency of the radiologist in busy practice environments.

In majority of radiology practices, the radiologist often does not see the patient, and the only point of contact for the patient is the technologist. The practice of communicating patient’s clinical information from the technologist to the radiologist vary from onsite documentation of patient history in the chart, attaching the referring physician’s note to the patient images, to direct site marking by the technologist before the examination. The musculoskeletal department at our institution adopted the policy of onsite marker placement at the site of concern, i.e. “most painful or only painful site” and/or palpable swelling. The purpose of this study was:

1. To evaluate the correlation of skin marker placement by the technologist to an important clinical finding, and
2. Study the impact of re-training of technologists on frequency of marker placement and correlation of marker site post-training with the underlying pathology.

## Methods

**Fig. 1:**
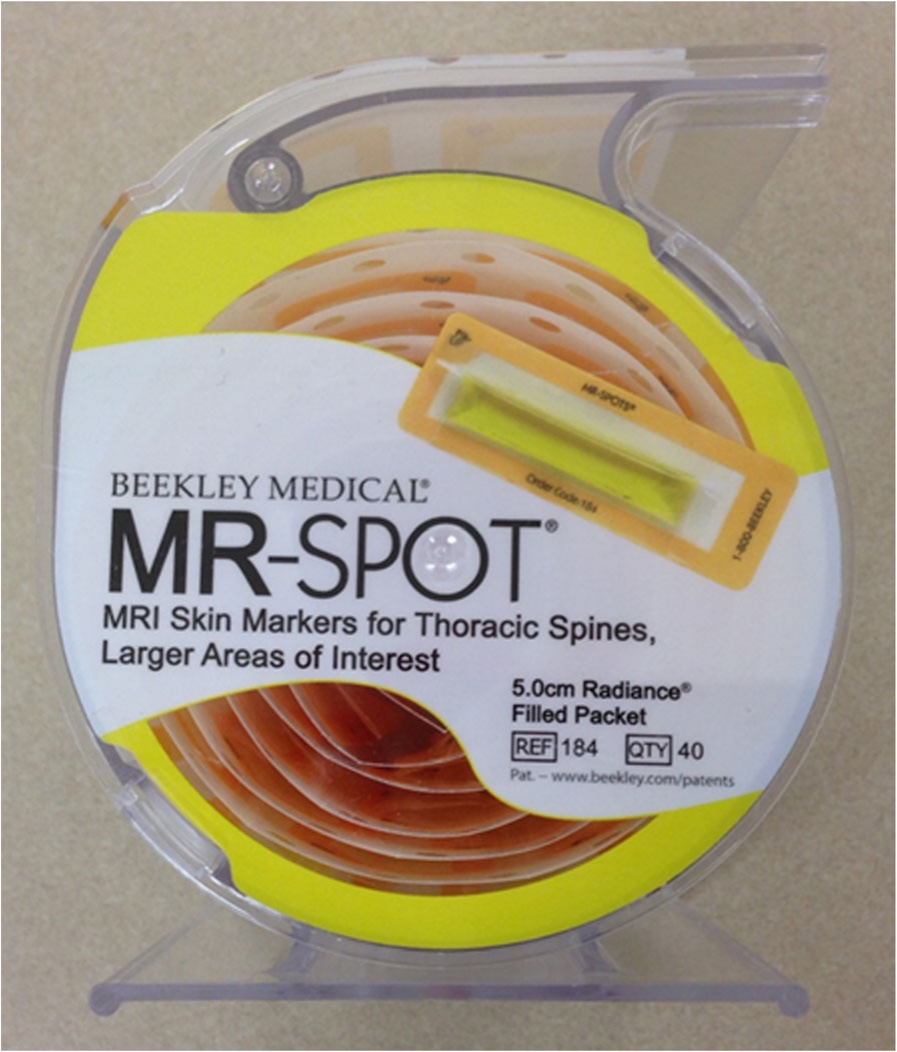
MR-SPOT MRI skin marker (Beekley Medical Corporation)

Institutional board review approved retrospective study was performed and informed consent was waived for this HIPAA compliant quality improvement (QI) project. 200 consecutive knee MRIs in patients were evaluated in two groups - before and after technologist training with regards to skin marker (MR-SPOT, 5.0 cm, Beekley Medical Corporation, Fig. 1) placement before the Knee MRI. Both pre-and post-training groups encompassed 100 scans each. Only left knee MRIs were included in the study to maintain consistency. The MRIs were evaluated for the frequency of marker placement by a musculoskeletal radiology fellow. The marker location in relation to the knee was categorized as: anterior, posterior, anterolateral, anteromedial, medial and lateral. The important imaging findings evaluated included: medial or lateral meniscus tear, medial or lateral collateral ligament tear, anterior or posterior cruciate ligament tear, high grade or full-thickness cartilage defects and moderate-large effusion. Patient demographics, presenting complaint and post-imaging treatment were also recorded. The imaging findings were classified as follows:

- corroborative and important, i.e. finding correlated with location of the skin marker. e.g. a medial meniscus tear in the setting of a medial marker.
- non-corroborative important, i.e. finding did not correlate with location of the skin marker, e.g. a lateral meniscus tear in the setting of a medial marker.
- other compartment important finding in corroborative cases, i.e. lateral and medial meniscus tears in a medial marker.
- diffuse abnormality, i.e. tricompartmental cartilage defects in both corroborative and –non-corroborative cases.

Differences among scans before and after the technologist training were assessed using chi-square test for categorical variables and t-test for continuous variables.

## Results

**Fig. 2:**
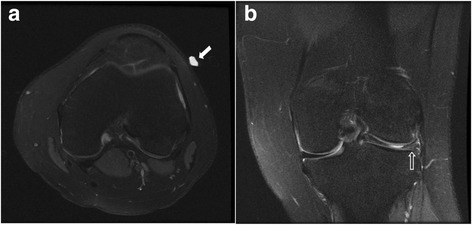
Lateral marker (solid arrow in **a**) and corroborative finding of bucket handle tear in the lateral meniscus (open arrow in **b**)

**Fig. 3:**
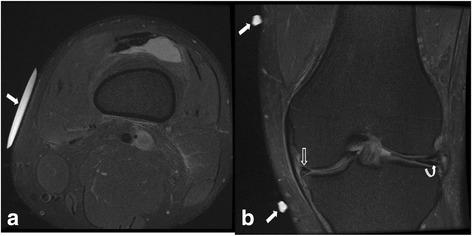
Medial marker (solid arrows) and corroborative finding of medial meniscal bucket handle tear (open arrow in **b**). Also note there is a lateral meniscal horizontal tear (curved arrow in **b**). Axial (**a**) and coronal (**b**) images

**Fig. 4:**
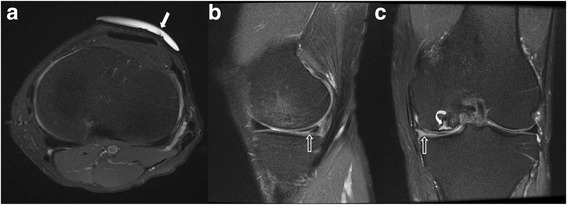
Anterior marker (solid arrow) and non-corroborative finding of medial meniscal tear (open arrows) and subchondral fracture (curved arrow). Axial (**a**), sagittal (**b**) and coronal (**c**) images

The results are summarized in Table 1. The patient demographics in both groups of scans were not statistically different (age *p* = 0.2 and sex *p* = 0.3). Chief complaint in majority of patients in both groups was non-specific pain (75% in pre-training group, 83% in post-training group, *p* = 0.16). Marker was observed in higher number of scans in the post training group (78% vs 60%, *p* = 0.00). Most common location of the marker was in anterior or anterolateral sites (32% and 34% cases, respectively). Corroborative and important findings were found to be higher, but not statistically different, in the post-training group (53% vs 38%; *p* = 0.57) (Figs. 2, 3 and 4). However, diffuse abnormalities were more prevalent in post-training group (31% vs 14%, *p* = 0.04), which may partially explain the statistical non-significance. Other associated findings in pre- and post-training groups (14% vs 13%) included medial meniscus tears (3 vs 7), lateral meniscus tears (3 vs 2), ACL tears (5 vs 1), MCL tear (1 vs 0), PCL tear (0 vs 1), and other compartment arthritis (2 vs 1). Majority of patients received conservative management (90%).

**Table 1:** Patient demographics and impact of marker placement in pre- and post-training groups

	Pre-Training Group	Post-Training Group	*P* value
Number of MRIs	100	100	
Age			0.2434
Mean	48.05	50.37	
SD	14.87	13.12	
Sex			0.3133
Female	56	63	
Male	44	37	
Clinician History			0.1649
Pain	75	83	
Other	25	17	
Marker Present			0.0059
Yes	60	78	
No	40	22	
Marker-Finding Correlation			0.5706
Yes	38	53	
No	22	25	
Diffuse Findings			0.004
Yes	14	31	
No	86	69

## Discussion

MRI-compatible skin markers have been used as a preoperative guidance tool for neurosurgeons in brain and spinal surgeries [3]. Fischer et al. described use of a special skin marker and inserted a nonmagnetic wire into the breast to achieve MRI-guided localization of suspected breast lesions. Repeat diagnostic MRI was performed to document the position of the wire tip relative to the lesion, to allow successful excision [4]. Besides commercially available markers, fish oil capsules have been used as MRI localization devices as a cost-effective alternative [5]. However, pre-scan application of skin marker in musculoskeletal settings has not been scientifically studied yet.

The present study shows that skin marker location highly correlates with important clinical findings, i.e. in upto 53% cases and thus, placement before knee MRI can potentially help the radiologist in producing clinically relevant reports. In routine reading of high volume of knee MRI studies and using structured template reporting, one may detect a number of imaging findings which could be unrelated to the patient’s symptoms, and may divert the attention of the radiologist from the area of interest. The clinical indication presented to the radiologist for joint MRI is usually non-specific (non-localizing) pain, as observed in the majority of cases in present study as well. Since there is limited clinical information available to the radiologist, skin markers can help in identification of pathology with increased confidence. Finding clinically correlated important internal knee derangement lesions in more than 50% cases by just using an external marker is a diagnostically relevant result. It should also be noted that in 47% cases, there were other findings, such as cruciate ligament injuries and non-correlated other compartment derangements. Therefore, comprehensive evaluation is still warranted during knee MRI reporting. However, the marker placement may help the radiologist rank the impressions more relevant to the clinical scenario and presentation.

Our study also shows that training of the technologists in application of knee markers at the area of interest, i.e. point of maximum pain or swelling, increases the frequency of marker placement. This cements the fact that, indirectly, active intervention of radiologist can keep up the interest of the technologists in quality improvement of imaging. Furthermore, the correlation of marker-important finding is also improved with technologist training and they become better at marking the clinically important site of abnormality.

We also found important imaging finding in 13–14% cases unrelated to the marked site. Therefore, although marking seems important and correlated finding should be put as the first impression, interrogation of all compartments of the joint remains essential.

There are some limitations to our study. First, this was a retrospective study and not a prospectively derived cohort. Second, we did not obtain inter-observer performance since formal reports were available for all the studies and this is the 1st exploratory study in this domain.

In future, similar studies in other joints, such as wrist, ankle, shoulder and hip could be performed to further validate the usefulness of marker placement. Time of imaging evaluation can be assessed with and without marker in detection of clinically important finding. A similar concept can be expanded to other imaging modalities, such as abdominal CTs, which are performed on a large scale, especially on call. It might help the reader to detect the important imaging finding quickly and consistently.

## Conclusion

Skin marker placement can aid in detection of clinically important imaging finding and technologist training aids in increased rates of marker placement and improved correlation. Therefore, marker placement can potentially improve the clinical relevance of knee MRI reports.

## Abbreviations

MRI: Magnetic Resonance Imaging
QI: Quality Improvement
